# HIV-associated neurocognitive disorder and HIV-associated myelopathy in a patient with a preserved CD4, but high viral load-a rarely reported phenomenon: a case report and literature review

**DOI:** 10.1186/s12879-020-05297-9

**Published:** 2020-08-05

**Authors:** Biniyam Alemayehu Ayele, Wondwossen Amogne, Lalise Gemechu

**Affiliations:** 1 Department of Neurology, College of Health Sciences, Addis Ababa University, Po Box 6396, Addis Ababa, Ethiopia; 2 Department of Internal Medicine, College of Health Sciences, Addis Ababa University, Addis Ababa, Ethiopia

**Keywords:** HIV-associated neurocognitive disorder, Seizure, cART, HIV-associated myelopathy

## Abstract

**Background:**

Despite widespread use of combination antiretroviral therapy (cART), HIV-associated neurocognitive disorder (HAND) and HIV-associated myelopathy (HAM) are not showing significant reduction in there occurrence. The HAM is a progressive myelopathy that often occur synchronously with severe form of the HAND in patients’ having advanced immunosuppression. However, co-existence of less severe form of the HAND and HAM in patient with relatively preserved CD4 cells is rarely reported clinical entity in post cART era.

**Case presentation:**

We report a 16-year old male, acquired HIV infection vertically, was on second line regimen because of virological failure since 3 years. His current CD4 lymphocyte count is 835 cells/uL with viral RNA level of 33,008 copies/mL. Currently presented with progressive forgetfulness, gait imbalance, and a frequent staring episodes without loss of postural tone. Neurological examination was pertinent for cognitive dysfunction with score of 6 on International HIV Dementia Scale (motor speed = 3, psychomotor speed = 2, and memory recall = 1). Lower limbs power is 4^−^/5, increased deep tendon reflexes, and unsteady gait. Brain MRI revealed diffuse both cortical and white matter T2 and FLAIR hyperintense lesions. Thoracic MRI showed abnormal T2 signal prolongation spanning from mid thoracic cord to conus. Electroencephalography study showed severe generalized slowing with evidence of focal dysrhythmia in bilateral frontotemporal regions. Unremarkable serum vitamin B 12 level (286 ng/mL). Virological failure with the HAND, HAM and seizure was considered. Dolutegravir +3TC + ATV/r regimen and valproate for seizure disorder was started. On 6 months follow up evaluation, he is clinically stable with significant improvement of his symptoms related to seizure disorders and modest improvement of his cognitive dysfunction, as he is now attending his school regularly. However, less improvement was observed reading his gait abnormality.

**Conclusion:**

This case supports the current understanding regarding the persistent occurrence of HIV-associated neurocognitive disorder and HIV-associated myelopathy even decades after introduction of cART. Therefore, it’s important to screen HIV+ patients for the HAND and HAM even if they have relatively preserved immunity. Because patient can be easily shifted to ART drugs with better CNS penetrating potential to achieve acceptable virological suppression level, to observe sound clinical improvement.

## Background

HIV-Associated Neurocognitive Disorder is considered the most frequent cause of dementia for people less than 40 years old [1]. Neurocognitive disorders have a complex classification, however its demonstration by clinical, neuropsychological and neuroimaging methods is important, because the HAND is considered an AIDS defining illness, its presence affects compliance with treatment and it has also been observed that antiretroviral drugs could revert its progress [2]. HIV-associated myelopathy usually seen in patients with advanced HIV/AIDS and often presented with progressive symptoms suggestive of chronic myelopathy. These symptoms includes: spastic lower limbs, gait abnormality, and bladder dysfunction [3].The thoracic segment of spinal cord is the most commonly involved part of the spinal cord. More specifically, the posterior and lateral columns of the spinal cord are vulnerable to white matter vacuolization neuropathological hallmark of the HAM. Moreover, its unique predilection for posterior and lateral columns of the spinal cord makes its diagnosis more difficult to differentiate from vitamin B12 deficiency which also has a close clinical and pathological resemblance to the HAM [4].

In the pre-cART era, the HAM usually developed in the advanced stages of the HIV infection with a progressive course until the patient’s death. Even though pathologic abnormalities of the HAM were found at autopsy in 22–55% of patients with advanced HIV infection [5, 6]. Up-to-date, there is no effective standard treatment of HIV-associated myelopathy, except symptomatic treatment of spasticity and bowel/bladder dysfunction. Nevertheless, few case reports suggested possible symptoms improvement of the HAM after initiation of cART [7, 8]. Even though, the severity and frequency of the HAND has decreased significantly in post cART era in developed world; the HAND is still a major cause of morbidity and mortality among HIV + patients living in Low and middle income countries [9]. According to one study from South Africa, 45% of HIV + youths met criteria for HIV-associated neurocognitive disorders [9].

Furthermore, simultaneous co-existence of the HAND and HAM in a single patient may have an additive effect in worsening patient quality of life and ultimately result in poor adherence to cART, which ultimately result in treatment failure. To our knowledge, this is the first case to be reported from Ethiopia. To this end, the aim of this case report is to shed a little light on the existing understanding of continuous occurrences of the HAND and HAM despite early initiation of cART and good immunological status, yet patient may have virological failure and may develop this AIDS defining major CNS complication.

## Case presentation

We report a 16-year old male, HIV + patient (vertical transmission) on second line combined antiretroviral therapy (cART), ABC/3TC + ATV/r regimen, with recent CD4 count of 835 cells/uL. He never had baseline viral load as the service was not available at his follow up area. He presented with progressive forgetfulness and gait imbalance over a period of 4 months. In addition, he has episodic loss of consciousness associated with sudden staring and automatism and hand tremor. He has blurring of visions and paresthesia of lower limbs. Past medical history was relevant for repeated admission for bacterial meningitis at a local hospital; after he presented with fever, headache, and neck pain. At a time he was investigated with CSF analysis, which was repeatedly suggestive of pyogenic meningitis. He was discharged after completion of full course of anti-meningeal dose of antibiotics. He denied any history of treatment for cryptococcal meningitis, no history of bowel or bladder dysfunction or history of upper limb weakness.

Neurological examination was pertinent for cognitive dysfunction with score of 6 on International HIV Dementia Scale (motor speed = 3, psychomotor speed = 2, and memory recall = 1). Patient’s functional status was not assessed formally by using standard tool. However, we have assessed his functional status by asking him about his daily activities, including home and school activities, he reported to have mild functional impairment, but able to live independent of anyone’s help for hid daily life. He fulfilled criteria for HIV-associated mild neurocognitive disorder (MND). Fully conscious and oriented with normal cranial nerves, lower limbs motor power was 4^−^/5, equivocal plantar responses, mildly increased tone, increased knee reflexes. Has an unsteady gait with flexed trunk and impaired tandem walk. He used to be on cotrimoxazole prophylaxis (CPT) but discontinued shortly because he couldn’t tolerate the drug.

**Fig. 1:**
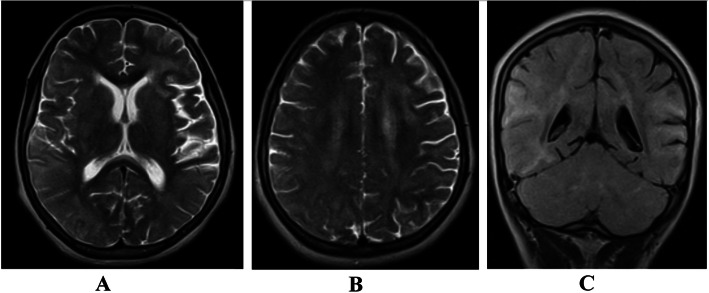
**a**, **b** Axial MRI showing diffuses both cortical and white matter T2 hyperintense lesions without mass effect (**a**, **b**). **c** Coronal FLAIR MRI sequence, showing hyperintense lesions on cortical and white matter areas of brain without mass effect

**Fig. 2:**
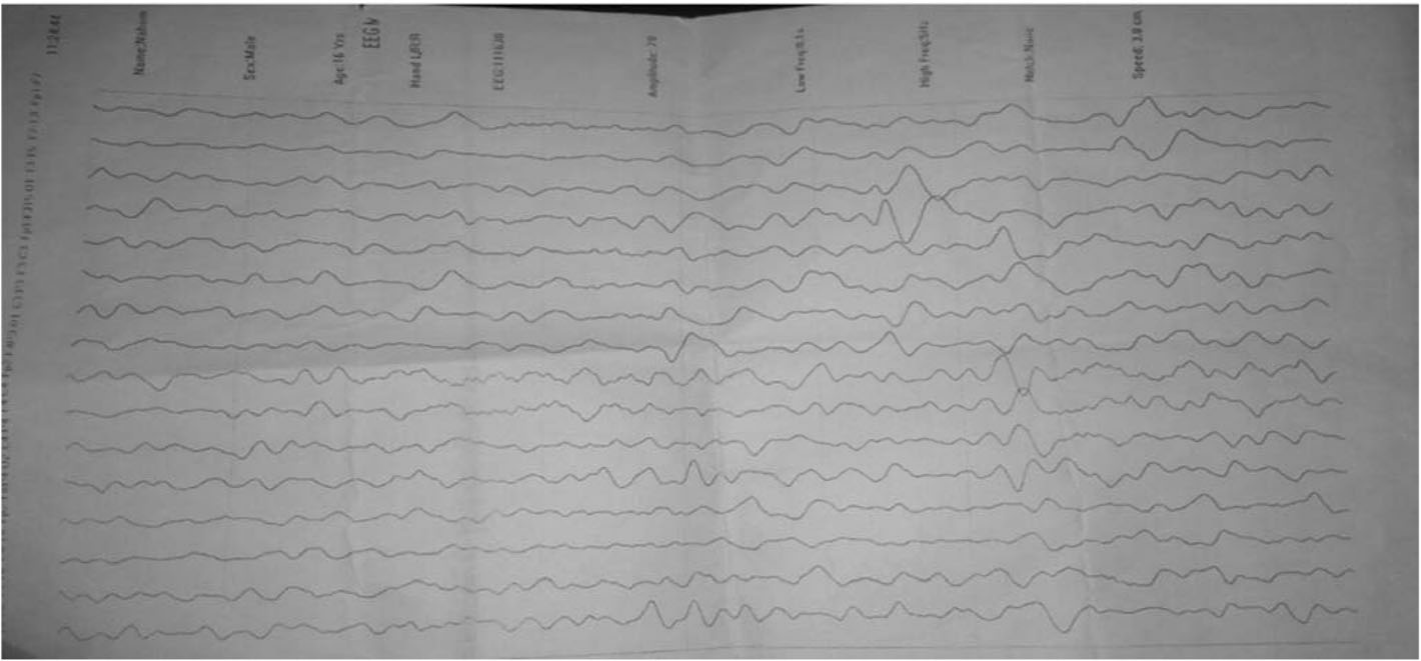
Electroencephalography (EEG) study showed severe generalized slowing with evidence of severe focal dysrhythmia around bilateral frontotemporal regions**,** indicating generalized encephalopathy related to the HAND

Routine hematological and serological tests were unremarkable. Viral load is 33,008 copies/mL (Table 1) Brain MRI revealed diffuses both cortical and white matter T2 and FLAIR hyperintense lesions, also minimally involving cerebellum, without mass effect (Fig. 1a, b, c). Thoracic MRI revealed abnormal T2 signal prolongation involving the cord, spanning from mid thoracic to conus, which is non-enhancing to contrast agent. Electroencephalography (EEG) study showed severe generalized slowing with evidence of severe focal dysrhythmia around bilateral frontotemporal regions (Fig. 2), indicating generalized encephalopathy related to the HAND.

**Table 1:** Patient’s Laboratory test, results and references

Laboratory tests	Results	Reference range
WBC	10,100 cells/mm^3^ N% 72	4500–10,000 cells/mm^3^
Platelet count	367,000 cells/mm^3^	150,000 to 450,000 cells/mm^3^
Hemoglobin	14.1 g/dL	14.0 to 17.5 g/dL
MCV	91.5 fL	79.4–94.8 fL
Urea	9 mg/dL	4.3–22.4
Creatinine	0.5 mg/dL	5.1–14
SGOT	46 U/L	0–35 U/L
SGPT	68 U/L	0–35 U/L
Total bilirubin	0.5 mg/dL	0.3–1.0 mg/dL
Direct bilirubin	0.09 mg/dL	0.1–0.3 mg/dL
GGT	43 U/L	9–50 U/L
VDRL	Negative	
HBSAg and anti HCV	Negative	
CD4 cells	835 cells/uL	500–1400 cells/uL
Viral load	33,008 copies/mL	< 50 copies/mL
Vitamin B 12	286 ng/mL	200–900 ng/mL

The patient was treated for pulmonary tuberculosis 5 years back (diagnosed based on chest X-ray) and completed his drug regimen and declared cured. On current spine MRI, there are no evidences of Pott disease, which is often radiologically characterized by presences of: wedged shaped vertebral fracture forming gibbus, paraspinous enhancing mass compressing spinal cord mainly in the lower lumbar region. Accordingly, our patient does not have any of these radiological features to make us suspect spine TB. Considering clinical presentations, examination findings, brain and spine MRI findings, EEG findings and higher viral load detection despite fairly maintained CD4 count; the patient was diagnosed as a case of virological failure + HIV-associated neurocognitive disorder + HIV-associated myelopathy + seizure disorder. The patient was started on integrase based regimen (Dolutegravir +3TC + ATV/r). In addition, valproate was started for seizure disorder and physical therapy for spasticity. On 1 month follow up, the patient has improvement in symptoms related to seizure disorders. However, no significant clinical improvement was observed regarding cognitive and gait abnormality. The latter symptoms may take longer time to result in observable clinical improvement.

## Discussion and conclusion

In the pre cART era, HIV-associated dementia occurred in up to 20% of individuals with acquired immunodeficiency syndrome (AIDS) and was almost always fatal [10]. Today, HIV-associated dementia is rare in the developed world, occurring in fewer than 5% of patients with HIV. This decrease is largely attributed to the wide spread cART use, as patients with HIV who are on ART perform better on neuropsychological testing than their ART-naïve counterparts [11]. Early identification of HAND is very crucial as un-treated HAND is associated with morbidity and mortality. Moreover, presences of the HAND may result in poor adherence to cART and ultimately paving ways to development of drug resistance [7]. In < 1% of patients, mainly in those with advanced HIV disease vacuolar myelopathy results in a spastic paraparesis with immobility and incontinence [9]. Few case reports indicated, early identification and initiation of cART may result in clinical improvement in patients suffering from the HAM [7, 8].

Our patient acquired HIV infection via vertical transmission from his parents who themselves are taking cART. His symptoms were progressive over 4 months, including insidious forgetfulness, lack of motive and gait abnormality, which are consistent with symptoms of HIV-associated neurocognitive disorder. Clinical features of HIV associated myelopathy were masked by features of the HAND, but increased lower limb tone, weakness and increased knee reflexes in addition to thoracic MRI indicating T2 hyperintensity supported our diagnosis of the HAM. Additionally, the HAM is said to be highly mimicked by vitamin B12 deficiency [4], but our patient had normal serum level of vitamin B12. To the author’s knowledge, this is the first case to be reported from Ethiopia. This case is unique not only because of the co-occurrence of the HAND and HAM, but it also supports the recent shift in clinical presentations of HAND, especially in post cART era. The HAND presenting with clinical features, traditionally thought to be related to cortical dementias, like Alzihmer disease [12]. Our patient predominantly presented with forgetfulness and seizure disorders, both indicating cortical involvement. So, we believe that this case report would add to the understanding of the HAND and HAM in our setup and open the door for future researches in an effort to understand these conditions.

In country such as Ethiopia, where issues of drug adherence and availability of less toxic and effective antiretroviral drugs are still a big challenge; it’s vital for practicing physicians to have high index of suspicion towards diagnosing the HAND and HAM. This should be true including for those HIV+ patients whom presented with clinical features suggestive of HAND, but have preserved cellular immunity (high CD4 cells count). This is especially relevant for children chronically infected with HIV as they have relatively normal CD4 counts. In addition, the clinical features of the HAND often mask prominent clinical features of the HAM. Therefore, it’s important to screen patients with HAND for possible coexistence of HAM. These practices will have a crucial public health importance, as early detection and optimal treatment of the HAND and HAM may result in clinical improvement, which will ultimately result in reduction in mortality and morbidity.

### Literature review on the HAND and HAM from African studies

Despite better access to cART compared to decades back, prevalence of HIV-associated neurocognitive disorder in Africa is still unchanged, and resulting in more poor adherences to cART. According to a meta-analysis of studies on prevalence of the HAND in HIV positive patients on ART for more than 6 months in seven countries in sub-Saharan Africa (Uganda, Zambia, Malawi, Botswana, Nigeria, Cameroon and South Africa), prevalence of neurocognitive impairment was more than 30% [13]. In Ethiopia, between years 2016 to 2019, total of 4 studies were published on prevalence of HIV-associated neurocognitive disorder among HIV + adults on cART at four different ART clinics located in the country by using International HIV Dementia Scale (IHDS) as screening tool. According to these studies, the prevalence of HIV-associated neurocognitive disorder was, 33.3% [14], 35.7% [15], 36.4% [16], and 67.1% [17].

According to one systematic review done on 19 studies conducted in Africa, HIV myelopathy was described in 3–16.9% of patients whom presented with myelopathy in Ethiopia and South Africa. Their diagnosis was based on clinical and neuroimaging evidences of HAM in patients with advanced immune suppression (CD4+ < 200 cells/ml) and after other potential mimickers are ruled out [18, 19]. Those countries without advanced neuroimaging facilities, diagnosis of HIV myelopathy was made in those patients who are HIV positive and presented with clinical signs and symptoms of myelopathy and no other causes of myelopathy were identified. In these studies, the proportion of myelopathy patients with HIV infection ranged from 14.1% in Nigeria [20], 30% in Ethiopia [18], to 50% in South Africa [21], these indirectly indicate the burden of HIV infection in these countries. Zenebe et al. [22], reviewed 130 patients admitted for spinal cord lesion between 1990 to 1993 to Tikur Anbessa Specialized Hospital, Addis Ababa, Ethiopia, to determine causes of spinal cord lesion. The authors reported, tuberculous spondylitis as the leading cause accounting for 35 (26.9%), and HIV associated myelopathies as the second common underlying etiology accounting for 22 (16.9%) of spinal cord disease.

## Data Availability

All data sets on which the conclusions of the case report based, to be available as spread sheets documents and available from the corresponding author on reasonable request from the editors.

## Abbreviations

MRI: Magnetic resonance imaging
cART: Combined antiretroviral therapy
HAND: HIV-associated neurocognitive disorder
HAM: HIV-associated myelopathy
HIV: Human immunodeficiency virus
AIDS: Acquired immunodeficiency syndrome
CD4: Cluster of differentiation
HAD: HIV-associated dementia
IHDS: International HIV Dementia Scale
EEG: Electroencephalogram
ART: Antiretroviral Therapy
3TC: Lamivudine
ABC: Abacavir
ATV/r: Atazanavir with Ritonavir booster
DTV: Dolutegravir
RNA: Ribonucleic Acid
